# Machine vs. human, who makes a better judgment on innovation? Take GPT-4 for example

**DOI:** 10.3389/frai.2023.1206516

**Published:** 2023-08-23

**Authors:** Mark Du

**Affiliations:** Department of Computer Science, National Taiwan University, New Taipei, Taiwan

**Keywords:** noise, creativity, artificial intelligence, large language models (LLM), GPT-4

## Abstract

**Introduction:**

Human decision-making is a complex process that is often influenced by various external and internal factors. One such factor is noise, random, and irrelevant influences that can skew outcomes.

**Methods:**

This essay uses the CAT test and computer simulations to measure creativity.

**Results:**

Evidence indicates that humans are intrinsically prone to noise, leading to inconsistent and, at times, inaccurate decisions. In contrast, simple rules demonstrate a higher level of accuracy and consistency, while artificial intelligence demonstrates an even higher capability to process vast data and employ logical algorithms.

**Discussion:**

The potential of AI, particularly its intuitive capabilities, might be surpassing human intuition in specific decision-making scenarios. This raises crucial questions about the future roles of humans and machines in decision-making spheres, especially in domains where precision is paramount.

## Introduction

Human decision-making is a complex process that involves a multitude of factors, including emotions, biases, and external influences. However, even when individuals strive to make rational decisions, they may still be prone to noise (Kahneman et al., 2021), which refers to irrelevant and random factors that can influence decision-making. This can have significant consequences in various fields, such as finance, medicine, and hiring, where noise can lead to suboptimal outcomes and undermine the effectiveness of decision-making processes. In contrast, artificial intelligence (AI) has the potential to make less noise than humans due to its ability to process vast amounts of data and apply logical algorithms to make decisions. There are some studies using GPT to examine the human mind, including semantic association (Digutsch and Kosinski, 2023), Theory of Mind (Kosinski, 2023), personality (Cao and Kosinski, 2023), decision making (Hagendorff et al., 2022), and even stock market (Lopez-Lira and Tang, 2023). This essay explores the concept of noise in human decision-making and examines how AI can help reduce noise and improve outcomes. By understanding the limitations of human decision and the benefits of AI, we can identify opportunities to enhance judgment processes and promote more effective and efficient outcomes in various domains.

In the book “Noise,” Daniel Kahneman argues that noise is a psychological factor that we don't want. On the other hand, creativity is something that we wish to do, which also involves a process of thinking in a different aspect. There's a lot of research focused on how to judge a new innovative idea (Grant, 2017). Creativity requires divergent thinking, which is a similar psychological concept to noise. When it comes to judging innovative ideas, it seems that people get both bias and noisy. According to “Noise,” Kahneman argues that the AI system is noiseless. But after GPT-4 (2023) came out, things get a little bit different. Chat GPT is an AI model using a probability algorithm, which means it could generate randomness, and of course, noise. However, this article investigates that even a noisy AI system is less noisy than human judgment.

In this article, we want to compare machine prediction and human judgment, there is already some research comparing both (Kleinberg et al., 2018). Even a simple model can outperform humans (Meehl, 1954). We also compare GPT-3, GPT-3.5, and GPT-4. GPT is constantly refreshing its algorithm, past research suggests that GPT will make some judgment mistakes like a human, but a new version of GPT shows that some of these mistakes have been corrected.

## Machine judgment: overcoming intuition

Noise is composed of lots of types of bias, one example is intuition. Humans make mistakes using intuition and create bias. Intuition bias refers to the tendency to rely too heavily on intuition or “gut feelings” when making decisions or judgments, rather than relying on objective evidence or systematic analysis. While some researchers suggest that gut feeling might be helpful when judging start-up projects (Huang, 2020), most behavioral economic research suggests the opposite. If the situation does not include emotion or social cues, gut feeling is not the best option to make decisions. We discover that GPT will not make the same mistake as humans.

One method to measure intuition is the cognitive reflection test (CRT; Frederick, 2005). A classic question is to ask how much a ball costs:

Prompt 1.1

*A bat and a ball cost $1.10 in total. The bat costs $1.00 more than the ball. How much does the ball cost?*

The correct answer is 5 cents, and the intuitive answer is 10 cents. Here is an example of how GPT-3.5 answer this question:

*Let's assume that the cost of the ball is x*.

*According to the problem, the cost of the bat is $1 more than the ball*,

*So: Bat cost* = *x* + *$1*.

*The total cost of the bat and ball together is $1.10:*

*Total cost* = *Bat cost* + *Ball cost $1.10* = *(x* + *$1)* + *x Simplifying the equation:*

*$1.10* = *2x* + *$1 $0.10* = *2x x* = *$0.05*.

*Therefore, the cost of the ball is $0.05*.

*So the correct answer is $0.05*.

As you can see, GPT-3.5 list the math formula to solve the question, instead of thinking about it intuitively.

Another problem is called Hospital Problem, we include GPT-3.5 and GPT-4′s answers in Appendix B, and they both answer correctly. GPT-3.5 will provide a mathematical calculation process, and GPT-4 will provide statistical theory.

If the probability question is deliberately confused, GPT-3.5 and GPT-4 will detect it, here's an example:

Prompt 1.2:

*A cab was involved in a hit-and-run accident at night. Two cab companies, the Green and the Blue, operate in the city. You are given the following data: 85% of the cabs in the city are Green and 15% are Blue. A witness identified the cab as Blue. The court tested the reliability of the witness under the circumstances that existed on the night of the accident and concluded that the witness correctly identified each one of the two colors 80% of the time and failed 20% of the time. Q: What is the probability that the cab involved in the accident was black?*

GPT-3 will answer the probability that the cab involved in the accident was black is 0.2. But GPT3.5 and GPT-4 will question the information. See another example in Appendix C.

Another type of question is called semantic illusion tests (Erickson and Mattson, 1981), questions that have inconsistencies that people usually ignore, are also created to assess inclinations for instinctive reactions. For example:

Prompt 1.3

*By whom was Galileo Galilei almost executed in the seventeenth century for claiming that the Earth is round?*

GPT-3.5 answer:

*It's important to clarify that Galileo Galilei was not persecuted for claiming the Earth is round. The Earth's roundness was well-known and accepted by educated people long before Galileo's time. Galileo was actually put on trial for his support of the heliocentric model, which proposed that the Earth revolves around the Sun, rather than the geocentric model, which stated that the Earth was the center of the universe*.

*Galileo was tried by the Roman Catholic Church, specifically the Inquisition, in 1633. He was found guilty of heresy for supporting the heliocentric model, which contradicted the Church's teachings at the time. Galileo was not executed, but he was placed under house arrest for the remainder of his life*.

As you can see, GPT-3.5 is aware of the wrong description in the question.

Another research also shows that GPT-3 can make the conjunction fallacy like humans when it comes to probability judgment (Binz and Schulz, 2023). But GPT-3.5 and GPT-4 can now correct this mistake. Here's the Linda problem (Tversky and Kahneman, 1983):

Prompt 2.1:

*Linda is 31 years old, single, outspoken, and very bright. She majored in philosophy. As a student, she was deeply concerned with issues of discrimination and social justice and also participated in anti-nuclear demonstrations*.

*Q: Which option is the most probable?*

– *Option 1: Linda is a bank teller*.
– *Option 2: Linda is a bank teller and is active in the feminist movement*.
– *Option 3: Linda is a member of the NRA*.

GPT-3.5 will choose Option 2, but if you inform GPT-3.5 of the base rate, it will give the right answer.

Prompt 2.2:

*But do you consider the base rate? Option A has a higher base rate*.

*GPT-3.5 Answer:*

*You are correct that the base rate should also be considered when making a probabilistic judgment. If we assume that the proportion of bank tellers in the population is much higher than the proportion of individuals who are actively involved in the feminist movement or the NRA, then Option 1: Linda is a bank teller, may actually have a higher probability than Option 2, despite the information provided in the passage. In this case, the most probable option would be the one with the highest base rate. Therefore, Option 1 could be a more probable option if we consider base rate information*.

GPT-4, on the other hand, will choose the right answer. Here's GPT 4 answer:

*Based on the information provided, we can deduce probabilities as follows:*

*Option 1: Linda is a bank teller. This option does not provide any information about her social or political beliefs, so it is neither supported nor contradicted by the given information*.

*Option 2: Linda is a bank teller and is active in the feminist movement. This option is more in line with the information provided about Linda's concern for social justice and her outspoken nature*.

*Option 3: Linda is a member of the NRA. This option is less likely given the information about Linda's participation in anti-nuclear demonstrations and her concerns for social justice*.

*Based on the information provided, Option 2: Linda is a bank teller and is active in the feminist movement, is the most probable option. However, it's important to note that this question is an example of the conjunction fallacy. The probability of two events occurring together (in “conjunction”) is always less than or equal to the probability of either one occurring alone. In this case, the probability of Linda being a bank teller (Option 1) should be higher than the probability of her being both a bank teller and active in the feminist movement (Option 2)*.

GPT-4 both considers the human intuition and probability base rate.

GPT can also answer the anchoring bias problem correctly now. In the initial experiment conducted by Jacowitz and Kahneman (1995), students were asked to estimate various quantities, including the length of the Mississippi River in miles. In a subsequent iteration, new students were given either an upper or lower limit for the correct answer (e.g., the Mississippi River is over 700 miles long), which were referred to as anchors. The researchers discovered that students were more likely to undervalue the actual quantity when presented with a lower anchor and overvalue it when given an upper anchor.

While previous (Jones and Steinhardt, 2022) shows that GPT-3 will show anchoring bias, GPT-3.5 can now answer the correct answer and question the wrong information in the prompt as well.

### Adjust top-p to 1.0 and temperature to 0

According to GPT-4 (2023), “top-p” (or “nucleus”) sampling is a method for generating text by selecting from a distribution of likely next words based on a probability threshold. The top-p sampling approach works by first calculating the cumulative distribution function of the probability distribution over the possible next words. Then, the model selects the smallest set of words whose cumulative probability exceeds a pre-defined probability threshold (usually denoted as p). This set of words is called the “nucleus” and represents the top-p probability mass. The model then samples from this set of words in proportion to their individual probabilities. In order to reduce noise, we want the top-p to be as high as possible. Temperature means how creative the response is, and in order to decrease noise, the temperature should be as low as possible.

## Literature review

### Creativity

Creativity has a similar concept to noise, both psychological constructs need divergent thinking, and some argue that decreasing noise will also decrease creativity (Kahneman et al., 2021).

When judging creative products, we can expect that there will be more noise than normal products, it is important to decrease standard deviation when forecasting these creative products.

In the research “Balancing on the creative highwire” (Berg, 2016), participants were asked to measure the novelty of creative products.

**Figure 1:**
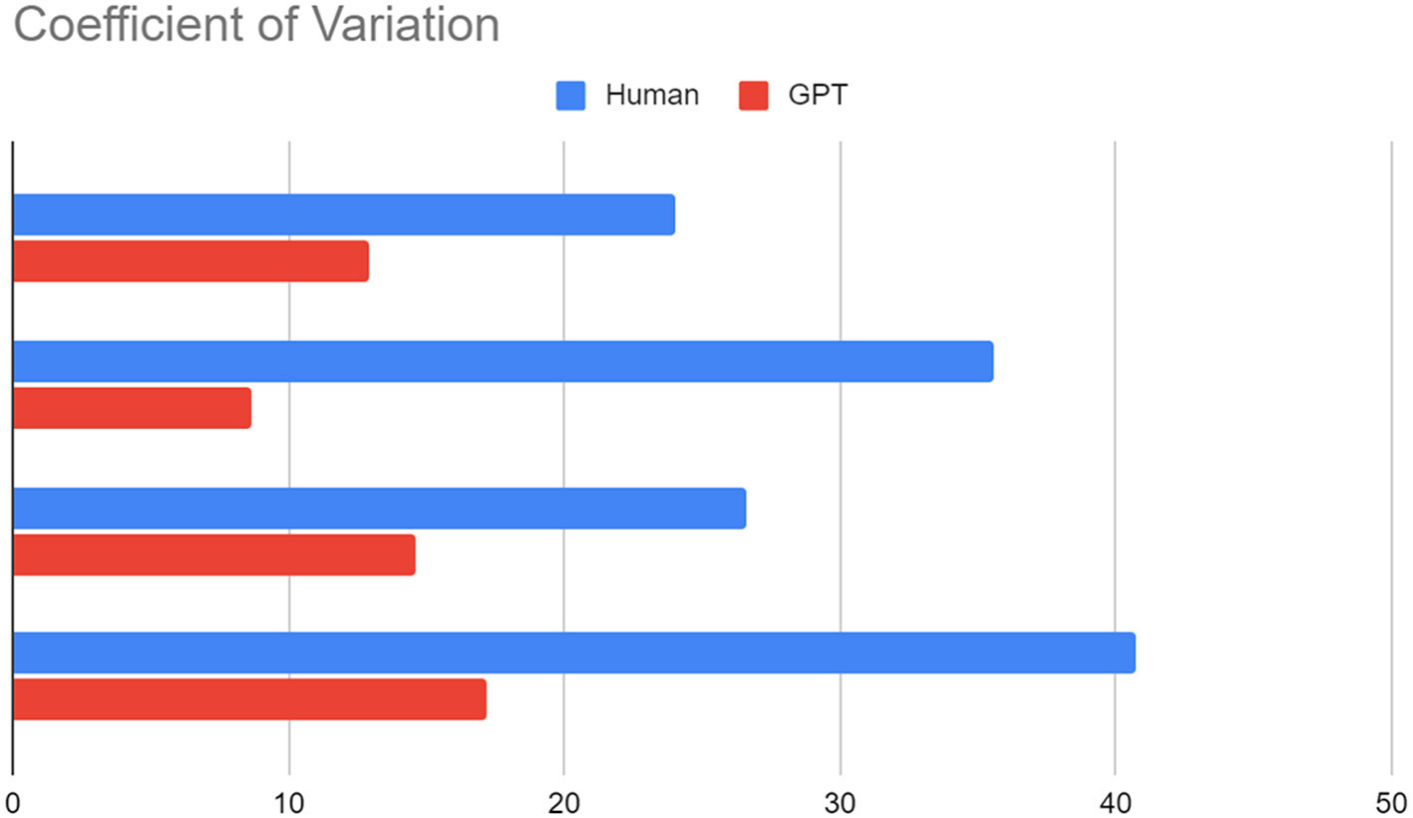
Experiment 1, four ideas rated by GPT and human.

We ask Chat GPT to do the same thing, using the method we mentioned above: Skip the comments, adjust the temperature to 0 and top-p to 1.0, we can see that GPT generates less standard deviation, and the coefficient of variation is lower (see Figure 1).

### Latent semantic analysis

As we mentioned above, even simple rules can beat human judgment (Meehl, 1954). Here we apply a simple rule called “Forward Flow” (Gray et al., 2019). It is a latent semantic analysis, a new method to measure creativity. LSA (Deerwester et al., 1990) computes the semantic similarity—or inversely, the distance—between two words based on the frequency of their co-occurrence within some corpora of text. The equation in this research is:


$$\left(\sum_{i=2}^{n}\frac{\sum_{j=1}^{i-1}D_{i,\;j}}{i-1}\right)/(n-1)$$

*D* is the semantic distance between thoughts, and *n* is the total number of thoughts within a stream.

It is really hard to quantify creativity, psychologists did not develop many psychological assessments to measure it, Forward Flow might be a stable way to measure novelty, namely the degree of novelty to use language.

Although past research shows that Forward Flow is negatively correlated to Kickstarter funding due to loss aversion (Tversky and Kahneman, 1974; Mueller et al., 2012; Tu, 2020), Forward Flow has changed its algorithm, past research focuses on the difference between words, and now Forward Flow focuses on the difference between sentences. This is closer to the traditional latent semantic analysis.

*This rule is noiseless*, and the simple rule is robust (Kahneman et al., 2021). When we try to predict something, using multiple variables such as multiple regression, will have an overfitting model problem, which might not consider the outliers. And creativity, by definition, often generates outliers.

Moreover, clinical judgment can produce information overweight, that is overlooking some variables. Creativity often contains lots of variables, it is easy to make people see something that really interests them, therefore giving a creative product a higher score than it should have.

The Forward Flow website is currently unavailable, so we use GPT-3.5 fun for us.

Prompt: Can you use forward flow to analyze the text for me?

Then Prompt: Do you know how to calculate the semantic distance for me? For example, grapes and apples have lower semantic distance because they are both fruit. On the other hand: ocean and table have larger semantic distances.

Then prompt: Can you calculate the overall semantic distance of words of an article below: “Text,” give me an average number.

### Human experiment

**H1:** Simple rules can outperform human.

**H2:** Machine learning can outperform simple rule.

#### Participants and procedures

This experiment included 40 participants in Taiwan recruited from a department of computer science at National Taiwan University. The experiment's objectives were to provide preliminary tests of H1 and H2. We randomly select eight Kickstarter programs to see the correlation between human rater and GPT. We use RCT to control the confounding variables.

We used the consensual assessment technique (Amabile, 1982, 1996). Raters were given the same broad definition of creativity as participants: “Overall degree to which the idea is both novel and useful,” which they rated using a 7-point scale (1 = “extremely low,” 7 = “extremely high”) (Berg, 2014, 2019). We measure the amount of funding the idea actually received.

We also use latent semantic analysis to generate a creativity score. As expected, *the standard deviation is 0*.

For GPT-4 the prompt is “judge how novel and useful the product is.”

## Results

**Figure 2:**
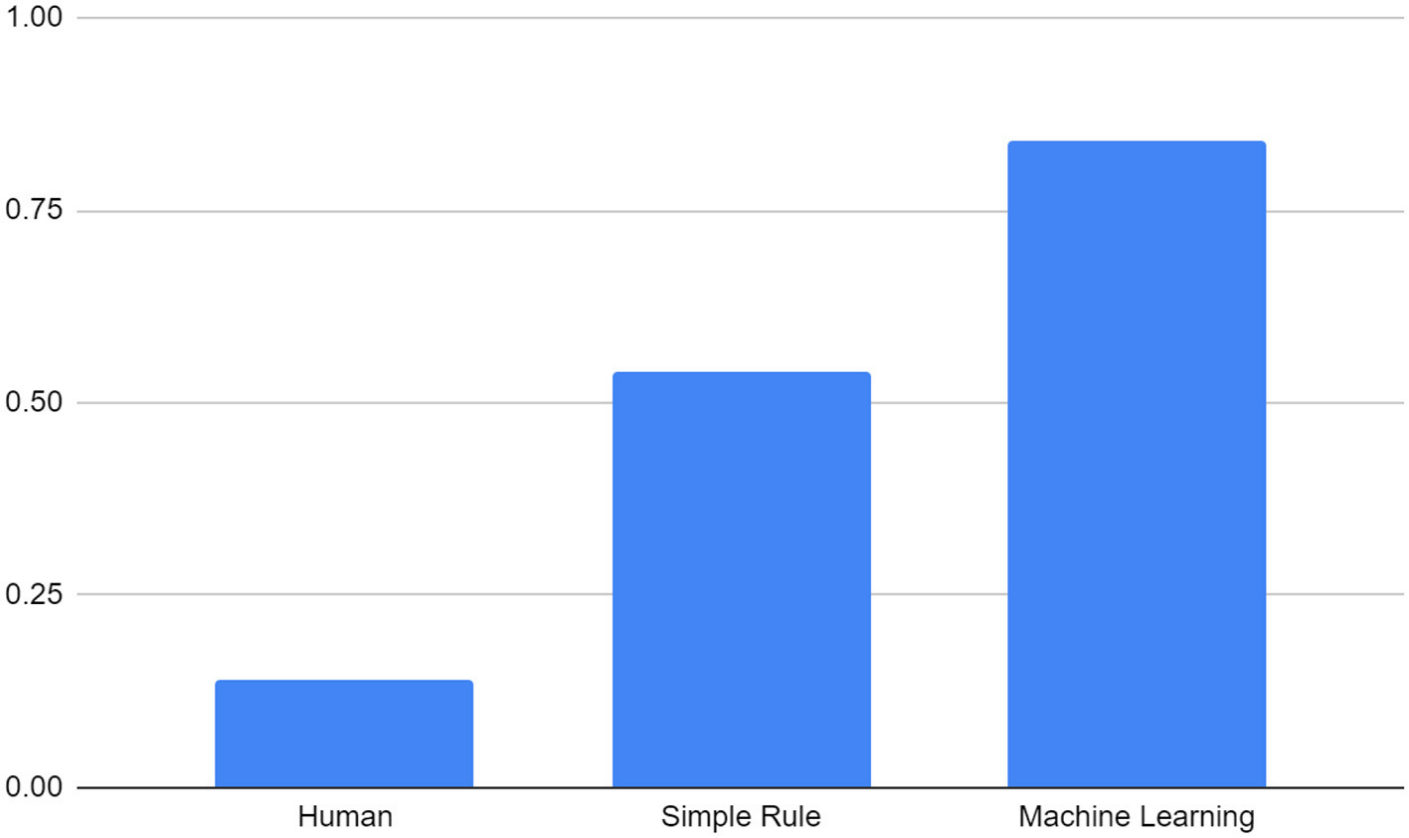
Pearson's r between Kickstarter funding and human, simple rule, and machine learning judgment.

The correlation between human raters' creativity score and Kickstarter fund is 0.14, latent semantic analysis is 0.54, and GPT is 0.84 (*P* < 0.05). This suggests that even simple rules can beat humans, and machine learning predicts even better (see Figure 2).

Regression analysis: The R-squared value of human rate is 0.02, the R-squared value of simple rule is 0.3, the R-squared value of GPT-4 is 0.713.

## Conclusion

Humans sometimes make intuition mistakes, but machines can overcome this right now.

GPT has improved very fast, GPT-3.5 and GPT-4 can answer cognitive reflection tests and causal reasoning questions much more precisely, and they can detect if the question itself has some flaws.

Next, even if a machine learning program is noisy, it is still less noisy than humans, but noise is not the only variable that improves prediction, we still have to consider bias.

### Future suggestion

The simple rule is noiseless, but it predicts less accurately than the machine learning algorithms, future research can examine how machine learning can detect the unseen pattern behind creative products, such as “broken leg problem.” People often consider machine learning biased against race, gender, and other minority, but since the algorithm become more and more complicated, the bias problem might be less severe than we think (Kleinberg et al., 2018; Logg et al., 2019; Kahneman et al., 2021). Machine learning contains lots amount of data, humans cannot possibly remember that much information, and GPT can provide similar product information from the internet. When answering such complicated problems as evaluating creativity, a large amount of data can prevent some heuristics (Simon and Newell, 1971).

## Data availability statement

The original contributions presented in the study are included in the article/Supplementary material, further inquiries can be directed to the corresponding author.

## Author contributions

The author confirms being the sole contributor of this work and has approved it for publication.
