# Supplementary material for: Machine vs. human, who makes a better judgment on innovation? Take GPT-4 for example
Source: Front Artif Intell. 2023 Aug 23;6:1206516. doi: 10.3389/frai.2023.1206516 (PMC10482032; doi:10.3389/frai.2023.1206516)
Supplement: Supplementary file 1 [file Data_Sheet_1.docx]

**Appendix A. Kickstarter program**

1. Hypershell is an innovative exoskeleton designed for everyday adventures, it is lightweight (1.8kg), and fast (maximum speed 20km/h). Hypershell is equipped with an artificial intelligence motion engine, allowing users to go further, climb higher, and carry more weight. The Hypershell is adjustable in size, weatherproof, and multisport adaptable for a variety of users and activities. The accompanying app can provide data insights and real-time control (speed monitoring, machine status, step count, mileage monitoring, and statistics) so that you can find your best state on every adventure.

2. LIFELONG Deodorant is a refillable, reusable, and washable deodorant applicator built to last. Available in three beautiful colors designed for both men and women. It features a one-of-a-kind magnetic lid and a knurled exterior for an industrial look and a comfortable grip. Plus, the crystal ball is removable, allowing you to customize the look of your deodorant with different colored crystal balls. Benefits to the planet include eliminating single-use plastic packaging, reducing shipping emissions, and no animal testing. Benefits for you include no harmful ingredients, no yellow sweat stains, and easy storage and portability. And comes with a magnetic lid to prevent leaks.

3. Flyweight - Major League Conditioning, Every Damn Day.

Flyweight is a versatile, configurable transformer that replaces eight different pieces of cardio and conditioning equipment. It allows full acceleration without the limitations of traditional equipment like dumbbells, kettlebells, medicine balls, and battle ropes. Flyweight focuses on adding power through acceleration, not weight, providing up to 130 lbs of resistance.

Flyweight's bi-directional resistance provides active acceleration and braking on both sides of the motion, working for each muscle group in perfect symmetry. It can be configured into different cardio machine setups, replacing eight tools like rowing machines.

4.Focusound™: The see-thru glass directional speaker

Focusing is an innovative directional sound technology that creates personal zones of sound without disturbing others. Transparent-designed speakers provide 360° surround sound. Focusound™️ No need for earphones, avoid tangled cables. Its Multiple SoundZone algorithm provides omnidirectional sound and spatial audio experience with adjustable angles for easy direction and angle control. Back Clip allows personalization without obstructing sound emission. Focusound DUO is equipped with two speakers to provide an enhanced audio experience up to 78db SPL.

5. Geo Wallet - World's 1st MagSafe Wallet with Full Find My

The ESR HaloLock Geo Wallet Holder is a product designed to solve the problem of lost or misplaced wallets. It magnetically attaches to your iPhone with a powerful built-in magnet and a suction force of 1,500 grams. The slim design holds 1-3 cards securely and also serves as a stable stand at various angles. The built-in rechargeable battery lasts up to 3 months on a single charge, and the wallet comes with a wrist strap for a better grip.

6. HANAGO: A NEW Musical Instrument Has Been Born!

The Hanago is a new and innovative stringed instrument that combines the key features of guitar and piano in one fret. It is easy to learn and helps users understand music theory easily. The instrument can be played in a variety of styles such as guitar, piano, or kalimba. Designed to be portable, durable, and lightweight, Hanago is perfect for anyone on the move. Made of beech and mahogany, this instrument produces a clear and smooth acoustic sound. Hanago is suitable for both beginners and experienced musicians and offers a variety of tutorials on its YouTube channel.

7. Duovox Ultra: Military-Grade Night Vision Monocular

The Duovox Ultra is a military-grade night vision monocular designed for outdoor adventurers, delivering 1080 FHD resolution and full-color imaging even in total darkness. Equipped with a Starvis CMOS sensor, built-in IR illuminator, high-sensitivity image sensor, and 7-layer glass lens, it can capture clear and detailed images in low-light conditions. It features a 5x optical zoom for sharp, distortion-free images. Duovox Ultra is tripod compatible, lightweight, and portable, perfect for nighttime exploration and photography.

8.NanoFoamer PRO - An Automatic Hands-Free Microfoam Maker.

The NanoFoamer PRO is an innovative device for making high-quality microfoamed milk at home, allowing users to enjoy cafe-quality drinks without expensive equipment. It speeds up the coffee-making process by heating and frothing the milk at the same time as the espresso is being prepared, ensuring perfect synchronization. Technically, the NanoFoamer PRO first preheats the milk to a given temperature while mixing it slowly. It then accelerates at a dizzying speed, mixing air into the milk. Finally, pull down and smash the merging bubbles with our clever rotating NanoScreens, which slow down to find the perfect balance.

**Appendix B Hospital Problem**

Prompt:

A certain town is served by two hospitals. In the larger hospital, about 45 babies are born each day, and in the smaller hospital, about 15 babies are born each day. As you know, about 50% of all babies are boys. The exact percentage of baby boys, however, varies from day to day. Sometimes it may be higher than 50%, sometimes lower. For a period of 1 year, each hospital recorded the days on which more than 60% of the babies born were boys. Q: Which hospital do you think recorded more such days?

- Option 1: The larger hospital.

- Option 2: The smaller hospital.

- Option 3: About the same (i.e., within 5% of each other)

GPT3.5’s answer:

Assuming that the percentage of baby boys is random and varies from day to day, we can use the binomial distribution to estimate the probability of having more than 60% of baby boys in a day for each hospital. Let's define success as a baby boy being born, and let's calculate the probability of having more than 60% success in a day, given the number of births in each hospital.

- For the larger hospital, with 45 births per day, the probability of having more than 60% of baby boys in a day is:

P(X > 0.6*45) = P(X > 27) ≈ 0.029

- For the smaller hospital, with 15 births per day, the probability of having more than 60% of baby boys in a day is:

P(X > 0.6*15) = P(X > 9) ≈ 0.18

Therefore, it is more likely that the smaller hospital recorded more days with more than 60% of baby boys since its probability of having such days is higher. So the correct answer is Option 2: The smaller hospital.

GPT-4’s answer:

This is due to the law of large numbers, which states that as the sample size increases, the sample mean converges to the true population mean. In this case, the larger hospital has a larger sample size (45 babies born each day) compared to the smaller hospital (15 babies born each day). With a larger sample size, the larger hospital is less likely to experience significant deviations from the expected 50% ratio of baby boys. The smaller hospital, with a smaller sample size, is more likely to have days with a higher percentage of deviation from the expected 50%, making it more probable for the smaller hospital to record more days with over 60% of babies born being boys.

**Appendix C. Confused Problem**

Prompt:

*Imagine a box that makes a sound if certain objects are placed on it. When I put a red object on it, the box made a sound. When I put a green object on it, the box made a sound. When I put a blue object on it, the box did not make a sound. When I put a yellow object on it, the box did not make a sound.*

*Q: I put a green and a yellow object on the box and it made a sound. Would it still have made a sound if I had not put the yellow object on it?*

*Q: Would the box make a sound if I only put a green object on it?*

*Q: But if there are two objects on the box, a green and a yellow one, and I remove the yellow one, then there would only be a green object on it. Why does that not turn on the machine?*

GPT-3’s answer:

A1: No, the box would not have made a sound if you had not put the yellow object on it.

A2: Yes, the box would make a sound if you only put a green object on it.

A3: The box would not make a sound if there is only a green object on it

GPT-3.5’s answer:

Q1: Yes, the box would still have made a sound if the yellow object had not been placed on it. Since the box made a sound when the green object was placed on it, and it made a sound when the green and yellow objects were placed on it together, we can infer that the green object is the one triggering the sound. The yellow object did not trigger the sound when placed alone, but it may have enhanced or modified the sound produced by the green object when they were both placed together. Therefore, the box would still have made a sound if only the green object was placed on it.

Q2: Yes, the box would make a sound if only a green object was placed on it, because it made a sound when the green object was placed on it alone.

Q3: It is possible that the yellow object has some property that, when combined with the green object, triggers the sound in the box, even though the green object alone is not sufficient to trigger the sound. The box may require both objects together to produce the sound, or the yellow object may modify the effect of the green object in some way that is necessary for the sound to be produced. Without further information about the properties of the objects and the mechanism of the box, we cannot determine why the green object alone does not trigger the sound when the yellow object is removed.

GPT-4’s answer:

Based on the information provided, it seems there might be a misunderstanding in the phrasing of the questions. Here are the answers based on the given information:

A1: Yes, because you mentioned that when you put a green object on the box, it made a sound. So, it would still have made a sound with only the green object.

A2: Yes, as per the information provided, the box made a sound when a green object was placed on it.

A3: Based on the information given, removing the yellow object would leave only the green object on the box, and since the box makes a sound with a green object on it, the box would still make a sound.
